# A Cluster Randomized Controlled Trial Evaluating the Efficacy of Peer Mentors to Support South African Women Living with HIV and Their Infants

**DOI:** 10.1371/journal.pone.0084867

**Published:** 2014-01-22

**Authors:** Mary Jane Rotheram-Borus, Linda M. Richter, Alastair van Heerden, Heidi van Rooyen, Mark Tomlinson, Jessica M. Harwood, W. Scott Comulada, Alan Stein

**Affiliations:** 1 Global Center for Children and Families, University of California, Los Angeles, California, United States of America; 2 Developmental Pathways to Health Research Unit, University of the Witwatersrand, Johannesburg, Gauteng, South Africa; 3 HIV/AIDS, STIs and TB, Human Sciences Research Council, Durban, KwaZulu-Natal, South Africa; 4 Department of Psychology, University of Stellenbosch, Matieland, South Africa; 5 Department of Psychiatry, Oxford University, Oxford, United Kingdom; Indiana University and Moi University, United States of America

## Abstract

**Objective:**

We evaluate the effect of clinic-based support by HIV-positive Peer Mentors, in addition to standard clinic care, on maternal and infant well-being among Women Living with HIV (WLH) from pregnancy through the infant's first year of life.

**Methods:**

In a cluster randomized controlled trial in KwaZulu-Natal, South Africa, eight clinics were randomized for pregnant WLH to receive either: a Standard Care condition (SC; 4 clinics; n = 656 WLH); or an Enhanced Intervention (EI; 4 clinics; n = 544 WLH). WLH in the EI were invited to attend four antenatal and four postnatal meetings led by HIV-positive Peer Mentors, in addition to SC. WLH were recruited during pregnancy, and at least two post-birth assessment interviews were completed by 57% of WLH at 1.5, 6 or 12 months. EI's effect was ascertained on 19 measures of maternal and infant well-being using random effects regressions to control for clinic clustering. A binomial test for correlated outcomes evaluated EI's overall efficacy.

**Findings:**

WLH attended an average of 4.1 sessions (SD = 2.0); 13% did not attend any sessions. Significant overall benefits were found in EI compared to SC using the binomial test. Secondarily, over time, WLH in the EI reported significantly fewer depressive symptoms and fewer underweight infants than WLH in the SC condition. EI WLH were significantly more likely to use one feeding method for six months and exclusively breastfeed their infants for at least 6 months.

**Conclusions:**

WLH benefit by support from HIV-positive Peer Mentors, even though EI participation was partial, with incomplete follow-up rates from 6–12 months.

**Trial Registration:**

ClinicalTrials.gov NCT00972699

## Introduction

Peer support is an important strategy for improving health outcomes for HIV, as it is for infant malnutrition, and adult diabetes [1]–[3]. Peer support not only improves health outcomes, but has the advantage of allowing tasks to be shifted from healthcare professionals to paraprofessionals [4]. With the number of healthcare personnel available in low- and middle-income countries (LMIC) unlikely to be sufficient to address HIV until the year 2050 [5], task-shifting to paraprofessionals is critical for supporting Women Living with HIV (WLH) to cope with their HIV-related stressors.

Peer support programs have been broadly diffused throughout Africa. For example, the Mothers-2-Mothers (M2M) program has linked newly diagnosed HIV-positive (+) WLH to Peer Mentors to Prevent Mother-to-Child Transmission (PMTCT) [6]–[8]. The M2M program is currently operational in 589 sites in 7 countries [8]. We are currently analyzing the M2M program's benefits of clinic-based HIV+ Peer Mentor support on post-birth outcomes of WLH and their infants [unpublished data]. This current paper extends that work and examines potential benefits of Peer Mentors over the first year of life.

South Africa has the highest total number of people living with HIV (5.7 million) [9], and 200,000 of the 3.2 million WLH are pregnant annually [9], [10]. The national HIV prevalence has stabilized around 11%, but 40%–60% of pregnant women in KwaZulu-Natal (KZN) are WLH [11]. PMTCT services have been routinely available in South Africa for more than 10 years [12], resulting in consistent improvements over time in the rates of participation in PMTCT [13]. Yet, uptake is similar to most of Africa, usually around 50% [14].

WLH face lifelong challenges, extending long after the completion of PMTCT. They must feed their infants using a single feeding method for 6 months (either breastfeeding or formula), preferably breastfeeding [15]–[17], maintain their own health, ensure that partners and family members are protected from HIV transmission, and cope with uncomfortable feelings (depression, anxiety) and stigma, as WLH decide how, when and to whom they disclose their HIV status [18]. Their caregiving, partnerships, social relationships, and daily stressors are likely to be impacted by their HIV status.

We have examined the post-birth outcomes of WLH in response to Peer Mentors, an Enhanced Intervention (EI) based on the key concepts of the M2M Program (i.e., placing HIV+ Peer Mentors in clinics to support newly diagnosed WLH) [18]. At about 1.5 months post-birth, WLH in the EI were significantly more likely to have infants with healthy height-for-age measurements than WLH receiving standard clinic care (SC). WLH in the EI also reported significantly fewer depressive symptoms and were more likely to ask their partners to test for HIV than SC WLH. However, WLH in the SC were more likely to adhere to antiretroviral medications (ARV) during pregnancy. Given the post-birth findings, we examine whether these improvements change from 6–12 months post-birth. In particular, previous studies have found that maternal depression affects infant health, including physical development (reflected in age-adjusted length and weight z-scores) [19]–[21]. We also continued to monitor HIV transmission-related behaviors, infant health, healthcare adherence, depression, and maternal social networks.

## Methods

Ethical approval was obtained from the Institutional Review Board of the University of California, Los Angeles (UCLA, G06-05-062), a Data Safety and Monitoring Board (DSMB), Community Advisory Board, and the Research Ethics Committee of the Human Sciences Research Council in South Africa (HSRC, REC 4/07/03/07). The protocol for this trial and supporting CONSORT checklist are available as supporting information; see Checklist S1 and Protocol S1.

### Setting

**Figure 1:**
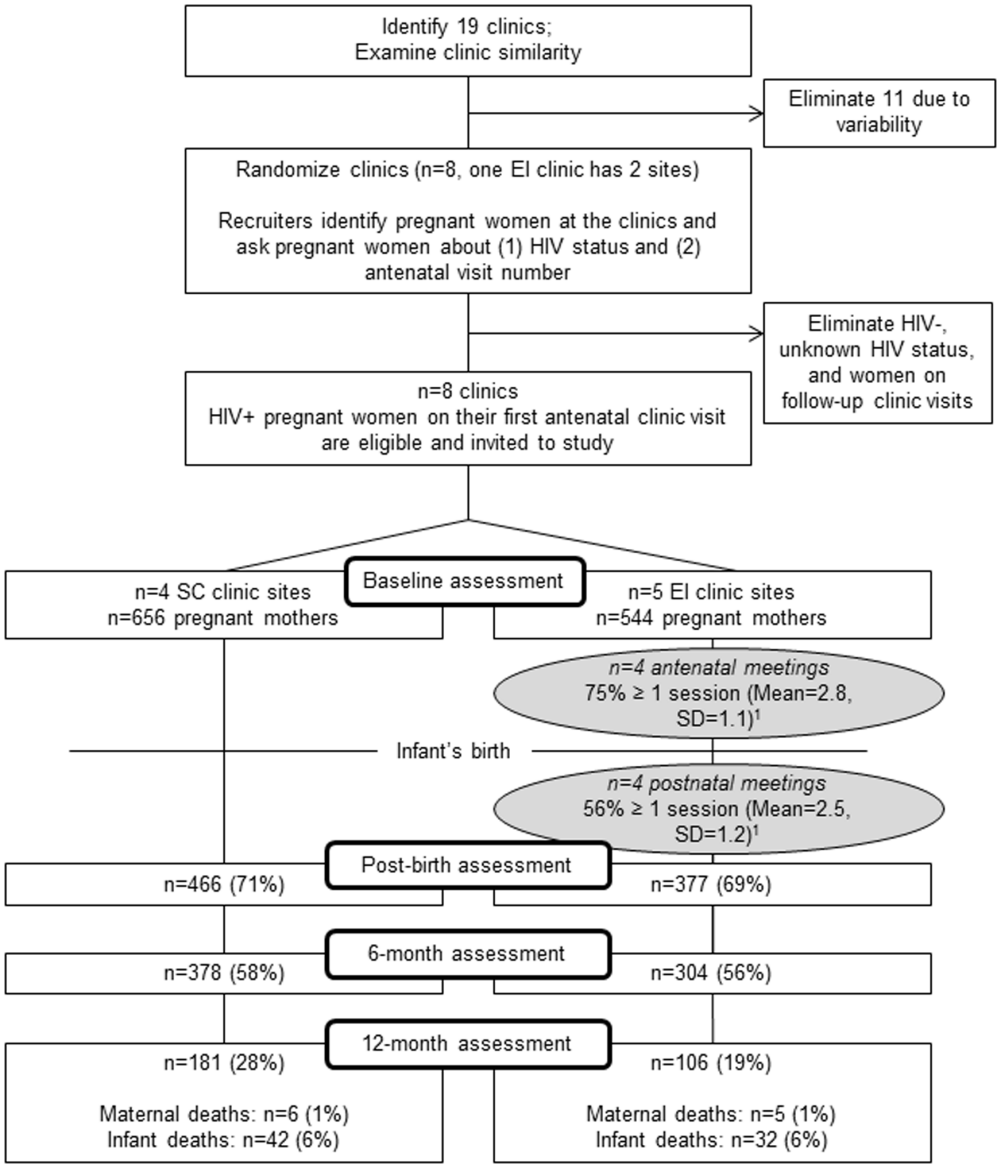
Movement of participants through the trial at each assessment point. SC = Standard Care; EI = Enhanced Intervention; ^1^ = This refers to mothers reassessed post-birth.

Eight clinics in KwaZulu-Natal were selected and matched on information collected from clinic surveys and observations regarding client load (i.e., 300+ pregnant women served annually), patient characteristics, the provision of both antenatal and child primary care services at the same site, rural/urban setting, and proximity to the main research site [18].On the basis of this information, the UCLA team randomized clinics to the Peer Mentor intervention Enhanced Intervention (EI), or the Standard Clinic Care Condition (SC). One clinic randomized to the EI had two sites. Figure 1 describes the flow of participants through the study design.

### Sample Size Calculations

Our power calculations were based on the randomization of eight clinics (within four matched clinic pairs) to either EI or SC, and assessments of WLH and their infants at least once between the ages of 6 weeks and 6 months post-birth. Sample size calculations were conducted to determine the minimum number of pregnant women that would need to be recruited per clinic to achieve 80% power to detect a standardized effect size of 0.25 between women from the four EI clinics and women from the four SC clinics on one overall summary measure, considering the anticipated base rate on each measure included in the index [18].

### Recruitment

All pregnant women were invited into the Peer Mentor program from July 2008 to April 2010, while in the clinic waiting rooms, and were informed again about the study by a nurse in their HIV testing session. A standard protocol was used across all clinics (see Protocol S1). However, we were only able to consistently collect data on all pregnant women entering the clinics after March 2009 (about half way through recruitment). Across the nine sites, recruitment into the Peer Mentor program ranged from 55% to 92% of all pregnant women testing seropositive for HIV; seven of the nine sites recruited more than 74% of eligible women. Overall, about 62% of WLH were successfully recruited into the study [18].

### Intervention Conditions

#### Standard clinic care (SC)

The Department of Health provided PMTCT services to all WLH in clinics across both conditions. PMTCT services included dual anti-retroviral (ARV) therapy for WLH during pregnancy and labor: nevirapine (NVP) for infants immediately post-birth and for the following six weeks until the infant HIV polymerase chain reaction (PCR) test results were obtained; referral for ARV among women with CD4 counts below 200 or WHO Stage 4 illness; and cotrimoxazole for HIV-exposed infants until HIV testing. Four antenatal clinic visits were recommended. Post-birth, WLH were offered HIV-related healthcare and infants had access to routine healthcare and immunizations, government child grants (R240/month, about 30 USD), and ARV if the infant tested HIV+ at 6 weeks using PCR tests. Maternal tuberculosis (TB) tests were recommended quarterly.

#### Enhanced intervention (EI)

On the day of their HIV diagnosis, WLH met with a Peer Mentor and were invited to attend eight meetings with peers, supplementing the standard clinic PMTCT programs. The meetings could be attended in any order and corresponded to routine antenatal and postnatal clinic services. The Peer Mentors were themselves WLH who had participated in a PMTCT program, were accepting of their HIV status, and willing to disclose their status to pregnant women. WLH in the EI were invited to meet with a Peer Mentor the day of diagnosis and to attend the series of group meetings that covered the following issues: 1) normalizing being a WLH; 2) establishing healthy daily routines without alcohol or smoking; 3) adhering to ARV medications and quarterly visits to an HIV clinic, monitoring CD4, disclosure of HIV status at delivery to healthcare providers; 4) obtaining a child support grant; 5) using a single feeding method, preferably breastfeeding for the infant's first six months, and not using traditional medicines during this time; 6) building and maintaining a social network; 7) consistent condom use, implementing universal precautions; 8) encouraging couple HIV testing and disclosure of HIV serostatus; and 9) bonding with her infant. Peer Mentors were trained in cognitive-behavioral skills, applying knowledge of PMTCT to daily life, building maternal skills, acquiring information in a manual, practicing each session serving as WLH, building skills using vignettes, supporting WLH to cope with their HIV status, and creating a personal statement about how the Peer Mentor adapted to her HIV status. Weekly supervision and regular debriefing was provided.

Overall, 87% of WLH in the EI re-assessed post-birth attended at least one intervention session: 75% attended at least one antenatal session (M = 2.8 sessions; SD = 1.1) and 56% attended at least one postnatal session (M = 2.5; SD = 1.2). Only 5% (n = 19) attended all eight sessions.

### Assessments

Trained research assistants aimed to interview WLH after their first antenatal visit, and post-birth at 6 weeks, 6 and 12 months. This report covers outcomes summarized between 6 to 12 months. All interview responses were recorded using commercially available survey software installed on low-end mobile phones [22]. The assessments covered the following domains: 1) HIV transmission-related behaviors, including disclosing HIV test results to partners, requesting partners test for HIV, using condoms during all sexual episodes, administering cotrimoxazole to their child, taking their child to receive HIV PCR testing at 6 weeks or 6 months and collecting the results, and using a single infant feeding method until six months of age; 2) Infant health status and bonding, including weight-for-age, height-for-age, and weight-for-height z-scores calculated using WHO age-and gender-based standards for growth (a z-score below −2 standard deviations was considered a serious growth deficit [23]), normal development according to WHO motor developmental milestones (using 50^th^ percentile ages: sitting without support (for infants at least 5.9 months old), standing with assistance (7.4 months), crawling (8.3 months), walking with assistance (9.0 months), standing alone (10.8 months), and walking alone (12.0 months)) [24], exclusive breastfeeding for at least 6 months, mother-infant bonding as assessed by the Postpartum Bonding Questionnaire (using a cut-off of 39 or below on the total score to indicate normal bonding) [25], and parenting stress as assessed by the Parenting Stress Index Short Form [26]; 3) Healthcare and health monitoring, including attending at least one postnatal clinic visit, maternal knowledge of CD4 cell count, and adherence to highly active antiretroviral therapy (HAART) medications; 4) Depression, as measured by the General Health Questionnaire (GHQ) [27] with a cut-off score of ≥7 for depressive symptoms; and 5) Social support, as measured by securing a child support grant and by the size of a mother's social network, calculated as the number of close friends and relatives multiplied by the frequency of contact in the past month.

Follow-up rates were lower than desired, 70% at post-birth, 57% at 6 months, and 24% at 12 months post-birth. About 24% (n = 290) were never re-assessed after the baseline interview; 19% (n = 230) completed only one follow-up assessment, and 57% (n = 683) completed two or more follow-up assessments. About 18% (n = 216) completed all assessments. Thus, because the follow-up rates were suboptimal, the analyses were conducted using the exact dates of the assessments.

### Data Analysis

Selection effects were examined between conditions at baseline and between those re-assessed and those lost to follow-up. To avoid multiple comparisons and to measure EI's overall effect on well-being, our primary analysis of the impact of the intervention compared WLH in EI and SC using a binomial test of the number of significant effects favoring EI among 19 measures. For outcomes assessed once, differences between conditions were tested at a one-sided upper-tail alpha = 0.025 using logistic random effects regressions adjusting for clinic clustering in SAS PROC GENMOD (version 9.2; SAS Institute Inc., Cary, North Carolina, USA). Models included an indicator variable for intervention status (1 = EI, 0 = SC). For outcomes assessed more than once, differences between conditions over time were tested at a one-sided upper-tail alpha = 0.025 using longitudinal logistic random effects regressions adjusting for clinic clustering and repeated measures on participants in SAS PROC GLIMMIX (version 9.2; SAS Institute Inc., Cary, North Carolina, USA). Models included the indicator for intervention status, time (months since the baseline assessment), and their interaction. The interaction term was the effect of interest.

We can expect 19*0.025 = 0.5 significant tests (i.e., less than one of 19) on average if there are no differences between EI and SC. If outcomes are independent, the probability that there are three or fewer significant differences is 99.9%, leading to a Type 1 error of 0.001. However, outcomes are likely to be positively correlated, which does not affect the expected number of positive tests, but does affect the variance of the number of positive tests. To study the effects of global positive correlation among all outcomes on the number of positive tests assuming no intervention effect, we treated each of our 19 tests as a normal z-test (z-statistics were assumed to come from an equi-correlated multivariate normal distribution) and simulated 40,000 trials of the number of significant outcomes, for z-tests having mutual correlations rho, with the value of rho running from 0 to 0.9 in steps of 0.1. We declared significance for z>1.96. Simulations were performed in R (version 2.11.1).

From the results, using a decision rule of rejecting the null of no EI treatment effect given 4 or more significant tests of 19, the type 1 error will stay below 0.05 no matter what the outcomes' correlations. We estimated the average absolute correlations among the outcomes using both Pearson (for “true dichotomies”, e.g. “Asked partner to test for HIV”) and tetrachoric correlations (for indicators created by dichotomizing continuous outcomes, e.g. height-for-age z-score≥−2), planning to use whichever method produced higher average absolute correlations.

Secondarily, we tested EI's impact on individual outcomes at a two-sided alpha = 0.05 using the regressions described above. We considered our secondary analyses to be exploratory and retained the model p-values in lieu of a multiple-testing adjustment.

## Results

### Sample Description

Table 1 describes the pregnant WLH at the baseline assessment. WLH were similar across conditions on each demographic and outcome measure. Women had an average age of 26.5 years (SD = 5.5). Most WLH (79.7%) had some secondary-level education, and 44.8% were employed. Although 82.7% of WLH reported having a recent sexual partner, only 21.3% were married or living with a partner, typical of cultural norms around marriage in the area. As shown in Figure 1, 1200 women were assessed at baseline.

**Table 1:** Baseline characteristics of sample (N = 1200), grouped by intervention condition: Enhanced Intervention (EI, N = 544) vs. Standard Care (SC, N = 656).^1^

	EI (N = 544)		SC (N = 656)		Total (N = 1200)		P-Value^1^
	n (%)		n (%)		n (%)		
Demographic Characteristics							
Mean age (SD)	26.5	(5.5)	26.5	(5.5)	26.5	(5.5)	0.936
Highest Education Level							0.873
No schooling/Grades 1–6 (primary)	81	(15.0)	108	(16.5)	189	(15.8)	
Grades 7–12 (secondary)	429	(79.3)	525	(80.0)	954	(79.7)	
Tertiary	31	(5.7)	23	(3.5)	54	(4.5)	
Married or lives with partner	102	(18.8)	153	(23.3)	255	(21.3)	0.656
Had a sexual partner, past 3 months	468	(86.2)	523	(79.7)	991	(82.7)	0.247
Employed	279	(51.3)	258	(39.3)	537	(44.8)	0.169
Living in formal housing	342	(63.0)	373	(56.9)	715	(59.7)	0.997
Water on site	370	(68.3)	392	(60.0)	762	(63.8)	0.783
Flush toilet vs. other types	309	(57.0)	291	(44.4)	600	(50.1)	0.583
Have electricity	387	(71.3)	554	(84.5)	941	(78.5)	0.132
Median days mother gone hungry past week (range)	0.0	(0–6)	0.0	(0–7)	0.0	(0–7)	0.611
Median days children gone hungry past week (range)	0.0	(0–6)	0.0	(0–5)	0.0	(0–6)	0.871
Maternal Health							
Any chronic illness	71	(13.1)	61	(9.3)	132	(11.0)	0.549
Tested Positive for TB during this pregnancy	3	(0.6)	19	(2.9)	22	(1.8)	0.088
Substance Use in Pregnancy							
Alcohol prior to pregnancy recognition	105	(19.3)	101	(15.4)	206	(17.2)	0.463
Alcohol after pregnancy recognition	32	(5.9)	27	(4.1)	59	(4.9)	0.186
Tobacco	38	(7.0)	69	(10.5)	107	(8.9)	0.742
Cannabis	7	(1.3)	4	(0.6)	11	(0.9)	0.274
Depression							
Moderate to severe depression (GHQ score≥7)	80	(14.7)	89	(13.6)	169	(14.1)	0.724
Depressed mood (EPDS score>12	210	(38.7)	227	(34.6)	437	(36.5)	0.501
Severe depressed mood (EPDS score>18)	53	(9.8)	48	(7.3)	101	(8.4)	0.435
Social Support							
Number of close friends and relatives times frequency of contact past month > median of 25^2^	297	(54.7)	289	(44.1)	586	(48.9)	0.084
HIV Disclosure and Partner HIV Status							
Told sexual partner about HIV status (N = 990)	190	(40.7)	181	(34.6)	371	(37.5)	0.721
Disclosed HIV status to friend (N = 679)	119	(38.5)	123	(33.2)	242	(35.6)	0.568
Asked current partner to test for HIV (N = 497)	165	(75.7)	212	(76.0)	377	(75.9)	0.947
Current partner HIV+ (N = 345)	97	(52.4)	93	(58.1)	190	(55.1)	0.770

1 . No significant baseline differences. EI and SC compared using random effects regression models, controlling for clinic clustering.

2 . Number of close friends/relatives: EI (median = 1, range = 0–15); SC (median = 1, range = 0–10); Total (median = 1, range = 0–15). Number of contacts with close friends/relatives, past month: EI (median = 22, range = 0–150); SC (median = 14.5, range = 0–262); Total (median = 17, range = 0–262).

There were several selection effects between WLH in the EI and the SC who were successfully re-assessed and those who were lost to follow up. Within the SC condition, re-assessed WLH were more likely to be married or living with their partner, employed, and have water on site and a flush toilet. Compared to EI mothers lost to follow-up, re-assessed EI mothers were older and less likely to have a chronic illness. There were no serious study-related adverse events.

### Outcome Measures

As shown in Table 2, EI out-performed SC on 4 of 19 outcomes, indicating significant overall benefits in EI compared to SC using the binomial test (correlation = 0.1, p = 0.006).

**Table 2:** Maternal and infant health and well-being outcomes from birth to 12 months post-birth (N = 913) grouped by intervention condition: Enhanced Intervention (EI, N = 405) vs. Standard Care (SC, N = 508).^1^

	EI (N = 405) n (%)		SC (N = 508) n (%)		Estimated odds ratio, EI vs. SC, (95% CI)^2^		2-sided p-value^2^	
HIV transmission-related behaviors								
Told sexual partner about HIV status^3^					1.00	(0.96, 1.03)	0.862	
Asked sexual partner to test for HIV^3^					1.05	(0.99, 1.10)	0.107	
Always used a condom^3^					1.03	(0.91, 1.16)	0.645	
Gave infant cotrimoxazole (6 months)	222	(99.6)	261	(98.5)	4.39	(0.54, 35.82)	0.167	
Took infant to 6-week or 6-month HIV PCR test and fetched results	206	(72.5)	247	(71.8)	1.07	(0.58, 1.97)	0.836	
One feeding method first 6 months: formula or breastfeeding	279	(91.8)	297	(78.6)	3.02	(1.20, 7.60)	0.019	*
Infant health status and bonding								
Weight-for-age z-score≥−2^3^					1.08	(1.01, 1.16)	0.035	*
Height-for-age z-score≥−2^3^					0.99	(0.90, 1.08)	0.759	
Weight-for-height z-score≥−2^3^					0.84	(0.76, 0.94)	0.002	x
Normal development according to WHO motor milestones (using 50th percentile ages)^3^					1.08	(0.95, 1.24)	0.238	
Breastfed exclusively for at least 6 months	57	(71.3)	50	(52.1)	2.38	(1.04, 5.44)	0.040	*
Brockington Postpartum Bonding Total Score ≤39 (lower score = better bonding, 12 months)	100	(98.0)	175	(98.9)	0.57	(0.06, 5.65)	0.631	
Parental Stress Index Score ≤ median of 85 (12 months)	50	(48.5)	95	(53.7)	0.84	(0.54, 1.30)	0.426	
Healthcare and health monitoring								
At least one postnatal clinic visit (6 months)	156	(63.2)	119	(44.9)	1.88	(0.84, 4.23)	0.127	
Mother knows CD4 cell count at 12 months	59	(59.6)	89	(53.9)	1.26	(0.75, 2.12)	0.373	
On average, missed no HAART medication doses, past week (12 months)	18	(94.7)	25	(78.1)	5.57	(0.48, 64.92)	0.170	
Depression								
Not depressed (GHQ<7)^3^					1.08	(1.03, 1.13)	0.002	*
Social support								
Receiving child support grant (1.5, 6, or 12 months)	251	(63.4)	345	(69.0)	0.78	(0.46, 1.31)	0.343	
Larger than the median baseline social network (>25)^3^					0.98	(0.96, 1.01)	0.250	

1 . Sample size reflects participants with at least one follow-up assessment. Sample sizes for each assessment (note that a variable may be among a subset of the full sample, have missing values, or both, leading to a smaller effective sample size): 1.5 months post-birth: EI (N = 377), SC (N = 466), total (N = 843); 6 months: EI (N = 304), SC (N = 378), total (N = 682); 12 months: EI (N = 106), SC (N = 181), total (N = 287).

2 . Random effects logistic regression, controlling for clinic clustering. Models include an indicator for intervention status.

3 . Longitudinal outcome. In addition to the indicator for intervention status, models include time (months since the baseline assessment) and an intervention-time interaction. The interaction term was the effect of interest. See Table 3 for observed values over time.

* EI significantly better than SC. 2-sided p-value<0.05 (binomial test: 1-sided, upper-tail p-value<0.025).

x SC significantly better than EI. 2-sided p-value<0.05.

Table 2 also summarizes the differences between EI and SC on individual outcome measures (note observed data for longitudinal outcomes are presented in Table 3). Compared to SC infants, EI infants were more likely to be fed using one feeding method (OR = 3.02, p = 0.019), to have a larger increase in weight-for-age z-score≥−2 between birth and 12 months post-birth (OR = 1.08, p = 0.035), and to be breastfed exclusively for at least 6 months (OR = 2.38, p = 0.040). EI mothers reported a larger decrease in depressed mood between baseline and 12 months post-birth (GHQ<7: OR = 1.08, p = 0.002) compared to SC mothers. However, infants in the EI had a smaller increase in weight-for-height z-score≥−2 between birth and 12 months post-birth compared to SC infants (OR = 0.84, p = 0.002).

**Table 3:** Observed data for longitudinal maternal and infant health and well-being outcomes (N = 1200) grouped by intervention condition: Enhanced Intervention (EI, N = 544) vs. Standard Care (SC, N = 656).^1^

	EI (N = 544)		SC (N = 656)	
	n (%)		n (%)	
HIV transmission-related behaviors				
Told sexual partner about HIV status^2^				
Baseline	190	(40.7)	181	(34.6)
1.5 months post-birth	298	(79.0)	339	(72.7)
6 months	10	(4.8)	15	(6.0)
12 months	5	(6.1)	9	(6.2)
Asked sexual partner to test for HIV				
Baseline	165	(75.7)	212	(76.0)
1.5 months post-birth	167	(77.3)	168	(64.6)
6 months	120	(87.6)	127	(79.4)
12 months	45	(84.9)	65	(72.2)
Always used a condom				
6 months post-birth	151	(79.1)	163	(75.8)
12 months	59	(73.8)	109	(76.2)
Infant health status and bonding				
Weight-for-age z-score≥−2*				
Birth	311	(83.6)	424	(92.2)
1.5 months post-birth	274	(81.8)	296	(79.6)
6 months	274	(95.1)	338	(93.6)
12 months	92	(96.8)	158	(96.3)
Height-for-age z-score≥−2				
Birth	199	(86.9)	363	(84.6)
1.5 months post-birth	76	(80.9)	45	(53.6)
6 months	53	(76.8)	45	(83.3)
12 months	24	(77.4)	20	(83.3)
Weight-for-height z-score≥−2^x^				
Birth	120	(62.5)	282	(71.0)
1.5 months post-birth	54	(71.1)	63	(85.1)
6 months	50	(76.9)	48	(90.6)
12 months	28	(75.7)	22	(100.0)
Normal development according to WHO motor milestones (using 50th percentile ages)				
6 months post-birth	109	(76.8)	108	(77.1)
12 months	37	(68.5)	72	(58.5)
Depression				
Not depressed (GHQ<7)*				
Baseline	463	(85.3)	567	(86.4)
1.5 months post-birth	357	(94.7)	409	(87.8)
6 months	285	(93.8)	336	(88.9)
12 months	99	(93.4)	152	(84.0)
Social support				
Larger than the median baseline social network (>25)				
Baseline	297	(54.7)	289	(44.1)
6 months post-birth	158	(52.0)	171	(45.4)
12 months	56	(52.8)	90	(49.7)

1 . Sample size reflects baseline sample. Sample sizes for each assessment (note that a variable may be among a subset of the full sample, have missing values, or both, leading to a smaller effective sample size): 1.5 months post-birth: EI (N = 377), SC (N = 466), total (N = 843); 6 months: EI (N = 304), SC (N = 378), total (N = 682); 12 months: EI (N = 106), SC (N = 181), total (N = 287).

2 . Only Baseline, 1.5-month, and 12-month data used in model. Model failed to converge and no estimates were produced when 6-month data was included.

* EI significantly better than SC. 2-sided p-value<0.05 (binomial test: 1-sided, upper-tail p-value<0.025).

x SC significantly better than EI. 2-sided p-value<0.05.

## Discussion

The current findings suggest that support from HIV+ Peer Mentors is efficacious in helping WLH engage in positive health behaviors for themselves and their infants for some tasks from 6–12 months post-birth. Symptoms of depression are lower over the first year of life. These findings are consistent with our initial results on maternal depressed mood at 1.5 months post-birth [unpublished data].

The effects of the EI on maternal depression are notable, particularly as similar research in other countries has found that maternal depression significantly impacts infant development negatively [28]. The results from the post-birth [unpublished data] and current analyses demonstrate that decreases in mental health symptoms are sustained over time.

The prevalence of depression during pregnancy among South African WLH is about 39% [29], similar to the high rates of depression in LMIC found in Pakistan [30] and elsewhere. Given the high prevalence of depression and the limited number of healthcare workers available in LMIC, task shifting to community health workers is imperative. Screening for and treating depression in WLH during antenatal or postnatal visits will benefit many WLH and their infants both immediately and in the long-term.

In addition to reduced depressive symptoms, EI WLH have a larger percentage increase in infants with healthy weight-for-age measurements compared to the SC WLH. WLH in the EI are also more likely to use only one feeding method for the first six months when compared to SC WLH. These results indicate that having a HIV+ Peer Mentor appears to have positive effects over the longer term.

While the benefits achieved in the study are important, there were other outcomes for which no differences were observed. Maternal stress and bonding with infants, partner prevention strategies, healthcare adherence, and social support were similar across conditions. Other research has found that the more sessions attended, the more likely that there will be improvements in health behavior [31]. Rather than only focusing on a clinic-based strategy, a home-based strategy may increase the impact of the intervention [32].

There are a number of limitations to this study. While the SC and the EI were similar on many variables, there were large baseline differences between conditions on employment status, housing, and maternal chronic illness. Controlling for clinic clustering, there were no significant differences between WLH in the EI and SC conditions, implying that clinic differences may be related to subcultural differences and geographic location. Living in a rural setting and being employed places many constraints on women's ability to attend clinic services. In many interventions, those most in need of intervention are most likely to attend [31], but this is less possible in rural southern Africa. At several clinics, employers would wait for several pregnant women to complete their antenatal care to return the women to the job site, which prevented women from staying for Peer Mentor sessions. The follow-up rates were lower than we hoped to achieve, and there were selection effects among the women who we were able to re-assess. We were able to follow women who had more resources initially. Finally, the low adherence achieved for the full eight meetings in the EI was a challenge. Regular attendance for clinic appointments, especially those with an additional intervention component, can be difficult for women to achieve [33]. Other challenges included migration of study participants after childbirth, and not wanting to visit the clinic regularly for fear of disclosing their HIV status. It may be that alternative delivery sites will need to be identified for supporting WLH; home visits may be one alternative.

This study was not powered nor anticipated to significantly impact the HIV status of infants. In this study only 2.5% of infants were HIV+, similar across conditions (EI, 2.6%; SC 2.5%), and similar to rates observed in other settings in South Africa [34]. We did not expect a significant reduction in serostatus outcomes for infants.

Findings from the current study suggest that even modest interventions—an average of four sessions –in addition to the standard care recommended for all pregnant women, can result in important longer-term impacts on overall maternal mental health and improved infant outcomes. These results suggest that utilizing WLH as community health workers in a peer mentoring model can be efficacious in promoting healthy infant development by teaching basic HIV-related skills and supporting concrete and sustainable positive behavior change.

## Supporting Information

Checklist S1 **CONSORT Checklist.**
(DOCX)

Protocol S1 **Trial Protocol.**
(PDF)
